# *Bifidobacterium bifidum* as an example of a specialized human gut commensal

**DOI:** 10.3389/fmicb.2014.00437

**Published:** 2014-08-21

**Authors:** Francesca Turroni, Sabrina Duranti, Francesca Bottacini, Simone Guglielmetti, Douwe Van Sinderen, Marco Ventura

**Affiliations:** ^1^Alimentary Pharmabiotic Centre and Department of Microbiology, Bioscience Institute, National University of IrelandCork, Ireland; ^2^Laboratory of Probiogenomics, Department of Life Sciences, University of ParmaParma, Italy; ^3^Department of Food, Environmental and Nutritional Sciences, University of MilanMilan, Italy

**Keywords:** *Bifidobacterium bifidum*, bifidobacteria, probiotics, genomics, microbiota

## Abstract

Bifidobacteria are considered dominant and for this reason key members of the human gut microbiota, particularly during the first one to two years following birth. A substantial proportion of the bifidobacterial population in the intestine of infants belong to the *Bifidobacterium bifidum* taxon, whose members have been shown to display remarkable physiological and genetic features involving adhesion to epithelia, as well as utilization of host-derived glycans. Here, we reviewed the current knowledge on the genetic features and associated adaptations of *B. bifidum* to the human gut.

## GENERAL FEATURES OF THE GENUS *Bifidobacterium*

The genus *Bifidobacterium*, a member of the *Bifidobacteriaceae* family, belongs to the *Actinobacteria* phylum (Stackebrandt and Tindall, 2000). Bifidobacteria are Gram-positive microorganisms with a high G+C DNA content, which were first isolated from feces of a breast-fed infant by Tissier in 1899, and then named *Bacillus bifidus* (Tissier, 1900). However, because of their morphological and physiological features, which are similar to those of lactobacilli, they were classified as members of the genus *Lactobacillus* for most of the 20th century and only starting from 1974 have been recognized as a separate genus (Buchanan, 2009). Currently, the genus *Bifidobacterium* is comprised of 48 different taxa, 40 of which have been isolated from the gastro-intestinal tract (GIT) contents of mammals, birds, or insects, while the remaining eight from sewage and fermented milk (Ventura et al., 2007a, 2009b, 2014). In addition, two bifidobacterial taxa, i.e., *Bifidobacterium crudilactis* and *Bifidobacterium mongoliense*, were isolated from raw milk cheeses (St Marcellin, Vercors area, France) (Delcenserie et al., 2013). Taking their different ecological niches into account and combining this information with a comparative analysis of their 16S rRNA sequences, as well as with other housekeeping genes (*clp*C, *dna*J, *xfp*, *dna*B, *rpo*C, and *pur*F), the various *Bifidobacterium* taxa can be clustered into six different phylogenetic groups, designated as the *Bifidobacterium adolescentis-*, *Bifidobacterium asteroides-*, *Bifidobacterium boum-*, *Bifidobacterium longum-*, *Bifidobacterium pullorum-*, and *Bifidobacterium pseudolongum-*phylogenetic groups (Ventura et al., 2007a). In this context, the *B. bifidum* species was shown not to fit in any of the above mentioned phylogenetic groups, thus suggesting the existence of unique and specific genetic features.

## BIFIDOBACTERIAL ECOLOGY

Bifidobacteria have been isolated from six different ecological niches. Bifidobacteria are widely distributed among animals whose offspring enjoy parental care, such as mammals, birds, social insects. Therefore, the reason for their ecological distribution may be due to direct transmission of bifidobacterial cells from mother/carer to offspring. Though bifidobacteria are commonly found in the animal gut, these microorganisms have also been found in three other ecological niches: (iv) human blood (*Bifidobacterium scardovii*), (v) sewage (e.g., *Bifidobacterium minimum* and *Bifidobacterium thermacidophilum*), and (vi) food products (e.g., *Bifidobacterium animalis* subsp. *lactis*). These atypical ecological niches are rather different from that of the GIT, and it is plausible that the identification of bifidobacteria in these environments is the consequence of contaminations from GIT (Ventura et al., 2007b).

Notably, bifidobacteria that belong to the species *Bifidobacterium animalis*, *Bifidobacterium adolescentis*, *Bifidobacterium dentium*, and *Bifidobacterium catenulatum* display a more cosmopolitan lifestyle (Lamendella et al., 2008).

*Bifidobacterium bifidum, Bifidobacterium breve*, and *Bifidobacterium longum* are specifically identified in the human gut and have been shown to represent part of the dominant bacterial members of the gut microbiota of breast-fed infants (Turroni et al., 2009a, 2012).

## BIFIDOBACTERIAL POPULATION IN THE HUMAN GUT

Bifidobacteria quickly colonize the intestine of infants during the first weeks of life due to selection by breast or formula milk, as confirmed by metagenomic analyses (Roger et al., 2010; Koenig et al., 2011). In breast-fed infants, *B. breve* is the dominant species, followed by *B. bifidum* and *B. longum* subsp. *infantis* (Turroni et al., 2012; Milani et al., 2013).

The fecal microbiota of infants is characterized by high levels of bifidobacteria (Harmsen et al., 2000; Turroni et al., 2012). The level of abundance of bifidobacteria within the human gut decreases with age, although ecological analyses based on FISH and metagenomic studies have estimated that their presence in the adult colon is around 4.3 ± 4.4% of fecal microbes (Eckburg et al., 2005; Mueller et al., 2006). In adult feces, *B. adolescentis* and *B. catenulatum* species are commonly detected, followed by *B. longum*. Recently, a study involving the isolation of bifidobacteria from human intestinal mucosal samples and fecal samples on selective media (i.e., by a culture-dependent method), followed by the sequencing of the 16S rRNA internal transcribed spacer (ITS) regions of individual isolates allowed a description of the biodiversity of the bifidobacterial population present in the human gut (Turroni et al., 2009a). This study identified the most abundant bifidobacterial species present in the human gut, being represented by *B. longum*, *B. pseudolongum*, *B. animalis* subsp. *lactis*, *B. adolescentis*, *B. bifidum*, *B. pseudocatenulatum*, and *B. breve*. Furthermore, this analysis highlighted that the distribution of bifidobacteria present in various human subjects underline both an inter-subject, and an intra-subject variability, as also confirmed through real-time quantitative polymerase chain reaction (qPCR) analyses of fecal samples from healthy adults (Guglielmetti et al., 2013).

Turroni et al. (2009b) evaluated the bifidobacterial composition of the human intestine by a microbiomic approach, through the analyses of five colonic mucosal samples from healthy adults. This work showed how each subject possesses a specific population of colonic bifidobacteria that is in agreement with the large inter-variability of the whole intestinal microbiota previously described (Eckburg et al., 2005; Palmer et al., 2007). These samples were dominated by 16S rRNA gene sequences closely related to the *B. pseudolongum* phylogenetic groups (74.5%), followed by members of the *B. longum* (17%) and *B. adolescentis* (8.5%) phylogenetic groups. Moreover, this culture-independent approach led to the identification of many novel bifidobacterial 16S rRNA gene sequences, which are presumed to represent as yet undefined novel bifidobacterial species.

As mentioned above, *B. bifidum* is among the first colonizers of the human gut, reaching high numbers in the infant gut, but also detected at low levels in adults (Turroni et al., 2012). Particularly, the analyses by qPCR of fecal specimens from healthy adults revealed the presence of *B. bifidum* in 76% of the analyzed samples (*n* = 82), with a mean log(10) number of cells per g of feces (±SD) of 6.5 ± 1.4, whereas the mean concentration of total bifidobacteria in the same samples was 8.6 ± 1.2 (Guglielmetti et al., 2013). Therefore, *B. bifidum* species is a frequent member of the intestinal bifidobacterial population in healthy adults.

## GENOMICS INSIGHTS INTO THE *B. bifidum* TAXON

Due to the availability of novel whole-genome sequencing approaches, research in molecular microbiology, in particular that related to pathogens, has undergone dramatic changes during the last decade. In recent times, genome-decoding efforts have also been directed towards gut commensals and probiotic bacteria such as members of the genus *Bifidobacterium* (Schell et al., 2002; Lee et al., 2008; Sela et al., 2008; Barrangou et al., 2009; Turroni et al., 2010; Bottacini et al., 2011, 2012; O’Connell Motherway et al., 2011). In 2009, a genomics-based discipline, named probiogenomics, was established, which aims to provide insights into the diversity and evolution of beneficial gut commensals, and to reveal the molecular basis for their adaptation and interaction with the mammalian gut (Ventura et al., 2009a). Thanks to these probiogenomics efforts, we have significantly expanded our understanding of the biology of gut microorganisms, such as bifidobacteria, and we have generated a large amount of data on metabolic capabilities, genetics, and phylogeny of these bacteria. Within the genus *Bifidobacterium*, just eight members of the *B. bifidum* species have had their genome sequenced out of 23 currently publicly available complete bifidobacterial genome sequences (NCBI source). Notably, of these eight, only three genome sequences are complete, while the remaining five B. bifidum genome sequences are still fragmented in multiple contigs. The genome size of a *B. bifidum* taxon ranges from 2.14 to 2.28 Mb, whereas such a genome displays a GC content of about 62%, which is in line with the average values described for genomes of members of the genus *Bifidobacterium* (Ventura et al., 2007b, 2012; Turroni et al., 2013b). The currently NCBI-deposited reference genome of the *B. bifidum* species belongs to the infant stool isolate PRL2010 (Turroni et al., 2010), which was sequenced and published in 2010. The *B. bifidum* PRL2010 genome is also similar (89% identity at nucleotide level) to that of *B. longum* subsp. *infantis* ATCC15697 (Sela et al., 2008), even though they belong to two distinct bifidobacterial phylogenetic clusters, perhaps reflecting the fact that they share a common ecological niche (Ventura et al., 2007a). A functional classification of the genes present in the *B. bifidum* genomes according to the Cluster of Orthologous Genes (COG) families allowed the identification of a large proportion (>10%) of genes assigned to the COG family of carbohydrate metabolism and transport, including genes predicted to be involved in mucin metabolism (see below).

*In silico* analyses of the *B. bifidum* PRL2010 chromosome identified candidate genes displaying a deviant G+C content and for this reason possibly acquired through Horizontal Gene Transfer (HGT), collectively referred to as the mobilome. The predicted PRL2010 mobilome includes a prophage-like element Bbif-1 (Ventura et al., 2010) and two loci encompassing type I Restriction/Modification (R/M) systems as well as a type III R/M system (Turroni et al., 2010). Moreover, the predicted mobilome encompasses a 19 kb DNA region, which appears to represent an integrated plasmid.

The evaluation of genome variability within members of the *B. bifidum* species was assayed by Comparative Genomic Hybridization (CGH) experiments and the use of *B. bifidum* PRL2010-based microarrays (Turroni et al., 2010). In this way genes from the sequenced *B. bifidum* PRL2010 strain were assessed for their presence or absence in the genomes of a set of seven *B. bifidum* strains, which included isolates from various infant fecal samples as well as the neotype of *B. bifidum* species. Interestingly, among the variable regions of the CGH map there are those predicted as the mobilome of PRL2010 as well as other genes predicted to be involved in bacterium–environment interaction, such as the genes specifying sortase-dependent pili (Turroni et al., 2010). In addition, analysis of the *B. bifidum* PRL2010 chromosome revealed novel insights into the metabolic strategies followed by this strain to degrade host-derived glycans, and in particular mucin-associated carbohydrates.

## THE GENOMICS OF MUCIN BREAKDOWN IN *B. bifidum*

Mucin represents the main component of the mucus gel layer that is covering the epithelial surface of the GIT (Podolsky, 1985). The main carbohydrate monomers found in mucin include *N*-acetylglucosamine, *N*-acetylgalactosamine, fucose, and galactose, which are sometimes linked to sialic acid and sulfate groups (Forstner et al., 1995). In addition, mucin contains salts, lipids, and many proteins, such as growth factors, lysozyme, defensins, immunoglobulins, trefoil factors, and several intestinal proteins (Johansson et al., 2008).

Among members of the genus *Bifidobacterium*, the ability to degrade mucin is a peculiar property of the *B. bifidum* species, which can hydrolyse the glycosydic bonds of mucin (Turroni et al., 2010, 2011) and utilize it as the sole carbon source (Guglielmetti et al., 2009).

*In silico* analyses of the genome sequences of PRL2010 revealed a relatively small set of genes dedicated to carbohydrate metabolism, which predominantly specified glycosyl hydrolases (GH), compared to other bifidobacteria (Turroni et al., 2011). Remarkably, about 60% of the identified GH-encoding enzymes from *B. bifidum* PRL2010 are predicted to be involved in the degradation of mucin-derived oligosaccharides, most of which are uniquely present in the *B. bifidum* chromosome relative to other currently available bifidobacterial genomes. Furthermore, according to the Carbohydrate Active Enzymes (CAZy) system (Cantarel et al., 2009), the *B. bifidum* PRL2010 genome is predicted to encode members of two carbohydrate-binding module (CBM) families, CBM32 and CBM51, that are suggested to bind to carbohydrate residues encountered in the mucin core structure. Notably, in bifidobacteria the genetic information corresponding to predicted CBM32 and CBM51 members were only detected in the genomes of *B. bifidum* (Turroni et al., 2011).

Additional insights into the behavior of *B. bifidum* PRL2010 to utilize mucin were obtained from functional genomics approaches, such as whole proteome profiling as well as transcriptomic investigations (Turroni et al., 2010). Several of the enzymes encoded by *B. bifidum* PRL2010 involved in mucin metabolism encompass extracellular enzymes, such as putative exo-α-sialidases, as well as a predicted 1,2-α-L-fucosidase and 1,3/4-α-L-fucosidase, and a putative cell wall-anchored endo-α-*N*-acetylgalactosaminidase (Ashida et al., 2008, 2009; Kiyohara et al., 2012). Additional PRL2010 encoded enzymes that are believed to be involved in mucin breakdown include four *N*-acetyl-β-hexosaminidases, and four β-galactosidases. The mucin-catabolic phenotype of PRL2010 is further facilitated by the presence of carbohydrate transporters belonging to various families, such as the ATP-binding cassette (ABC-type), phosphoenolpyruvate phosphotransferase system (PEP-PTS) and major facilitator superfamily (MFS).

The genome of *B. bifidum* PRL2010 encompasses a DNA region spanning eight genes, which encode enzymes for the breakdown of galacto-*N*-biose that forms one of the core structures of mucin-oligosaccharides. In this context it is believed that *B. bifidum* PRL2010 accesses mucin-derived galacto-*N*-biose by the action of extracellular enzymes like the exo-α-sialidases and 1,2-α-L-/α-1,3/4-fucosidases, which perform de-sialidation and de-fucosylation, respectively, of mucin-derived oligosaccharides to facilitate further breakdown by the action of other enzymes encoded by PRL2010 such as the lacto-*N*-biosidase and endo-α-*N*-acetylgalactosaminidase.

Comparative genomics analyses involving all other currently available *B. bifidum* genomes revealed a high conservation of the predicted genetic arsenal involved in mucin breakdown (Turroni et al., 2011). Thus, it is tempting to conclude that mucin metabolism in PRL2010 is a genetic feature of most if not all members of the *B. bifidum* species rather than of a unique strain. Mucin degradation, which is expected to reduce the mucin layer and consequently reduce the protective barrier covering the intestinal mucosa, is generally considered as an undesirable event. However, one may also consider breakdown as an evolved “host-settler mechanism.” In fact, mucin production in the GIT normally initiates only several months after birth and reaches its mature level at about 12 months (Hooper et al., 1999). Interestingly, mucin breakdown activity as operated by *B. bifidum* could trigger the secretion of additional colonic mucin, thus increasing the thickness of the total amount of mucus layer covering the gut and so reinforcing the epithelial barrier function, which constitutes an important feature especially in those subjects affected by irritable bowel syndrome (Caballero-Franco et al., 2007). The capacity to efficiently use mucus is a typical feature also of *Akkermansia muciniphila*, a human intestinal species that has been associated with healthy intestines and disease prevention (Guglielmetti et al., 2008b).

## PILI PRODUCTION BY *B. bifidum* AS KEY HOST–MICROBE EFFECTOR MOLECULES

Non-flagellar appendages decorating the microbial cell surface were identified in bacteria in the early 1950s and since then the molecular data about their assembly, composition, and function has greatly expanded, especially for pathogens (Telford et al., 2006). In this context, these extracellular structures are considered crucial in the initial establishment of pathogens inside the host and are consequently considered key effector molecules in pathogenesis. However, their identification in bifidobacteria was only very recently established (Foroni et al., 2011; O’Connell Motherway et al., 2011). It has been shown that bifidobacterial genomes belonging to *B. bifidum*, *B. longum* subsp. *longum*, *B. adolescentis*, *B. dentium*, *B. animalis* subsp. *Lactis*, and *B. breve* contain one to seven predicted sortase-dependent pilus gene loci, each of which are predicted to encode one major pilin subunit (represented by FimA_PRL2010_ or FimP_PRL2010_ for the *pil*2_PRL2010_ and *pil*3_PRL2010_ clusters, respectively) plus a minor pilin subunit (represented by FimB_PRL2010_ and FimQ_PRL2010_ for the *pil*2_PRL2010_ and *pil*3_PRL2010_ clusters, respectively), as well as a so-called sortase, a protein specifically dedicated to covalently assemble these pilin subunits (Foroni et al., 2011).

Very recently, the four *B. bifidum* genome sequences that are currently publicly available have been screened for sortase-dependent pili leading to the identification of three loci (Turroni et al., 2013a). Of these three identified loci, only two were shown to be genetically intact whereas the third appeared to be non-functional due to a frameshift within the coding region of the gene encoding major pilus subunit (Turroni et al., 2013a). When FimA_PRL2010_ was compared to FimA homologs encoded by other *B. bifidum* strains, their amino acid sequences were shown to display much higher variability compared to the FimP homologs (Turroni et al., 2013a). Furthermore, FimA_PRL2010_ includes a CnaB-type domain that is known to serve as a stalk in binding to components of the Extra Cellular Matrix proteins of the host, such as fibronectin, collagen types I to XV, and laminin (Deivanayagam et al., 2000).

Transcriptomic investigations performed on *B. bifidum* PRL2010 upon colonization of mice as well as upon contact with human cell lines, demonstrated a clear transcriptional up-regulation of those genes encompassing two sortase-dependent pili, named *pil2* and *pil3* (Turroni et al., 2013a). Heterologous expression of the pilus-encoding genes corresponding to Pil2 and Pil3 in the non-piliated, Gram-positive host *Lactococcus lactis* showed that both types of pili are modulating the adhesion to human enterocytes through extracellular matrix (ECM) proteins and bacterial aggregation. ECM deglycosylation provoked a dramatic reduction in PRL2010 pili-mediated binding ability compared to untreated ECM (Turroni et al., 2013a), pointing that N- and/or O-linked glycoproteins are involved in adhesion of PRL2010 pili to ECM. Furthermore, carbohydrate binding competition experiments demonstrated that mannose and fucose act as potential receptors for Pil2 of *B. bifidum* PRL2010 in a fashion that is reminiscent of that previously described for other enteric bacteria (Farfan et al., 2011), whereas the putative binding partners for Pil3 appear to encompass a larger set of carbohydrates (Turroni et al., 2013a).

Recombinant piliated *L. lactis* cells were also shown to evoke a higher tumor necrosis factor alpha (TNFα) response during murine colonization compared to their non-piliated parent, indicating that *B. bifidum* PRL2010 sortase-dependent pili not only contribute to adhesion but also display immunomodulatory activity (Turroni et al., 2013a). Triggering TNFα production by pili synthetized by *B. bifidum* PRL2010 may represent an intriguing feature of this species as one of the first colonizers of the human gut (Turroni et al., 2012). In this context, it is worth mentioning that cytokines belonging to the TNFα superfamily are not only linked to the occurrence of inflammatory diseases (Yasutake et al., 1999), but also exert a major role in the rejection of tumors and the response to infections (Wajant et al., 2003; Lebeer et al., 2010). Furthermore, the induction of TNFα may be crucial for the initiation of cross-talk among immune cells without provoking any inflammation or detrimental effects (Galdeano et al., 2007).

## OTHER HOST RESPONSE EFFECTOR MOLECULES ENCODED BY *B. bifidum*

Recently, the murine lytic enzyme TgaA encoded by *B. bifidum* MIMBb75 has been molecularly characterized (Guglielmetti et al., 2014b). This peptidoglycan-degrading enzyme contains two active domains, i.e., a lytic murine transglycosylase and a cysteine histidine-dependent amidohydrolase/peptidase (CHAP) domain and was demonstrated to exert immunomodulatory effects (Guglielmetti et al., 2014a). The TgA-encoding gene does not appear to be widely distributed among the currently available *B. bifidum* genomes and thus represents an example of a strain-dependent gene (Guglielmetti et al., 2014b).

Other *B. bifidum* proteins involved in host interaction are represented by the surface lipoprotein BopA, which was originally described to be involved in adhesion to intestinal epithelium (Guglielmetti et al., 2008b; Gleinser et al., 2012). However, recently the role of BopA in the adhesion of *B. bifidum* was reassessed and, in contrast to what was published earlier, the strong adhesion of *B. bifidum* to epithelial cell lines is mainly BopA-independent (Kainulainen et al., 2013).

Within the surface proteins encoded by *B. bifidum* strains responsible of adhesion to the human intestine, the transaldolase Tal of *B. bifidum* A8 has been also proposed (Gonzalez-Rodriguez et al., 2012). Notably, such protein has been shown in modulating the adhesion to mucin as well as to promote bacterial aggregation, thus could act as a key colonization factor in driving the establishment of *B. bifidum* cells in the human gut.

## IMMUNE RESPONSE OF *B. bifidum* STRAINS

Members of the *B. bifidum* species have been claimed to exert an important role in the evolution and maturation of the immune system of the host, which is still undeveloped at birth (Lopez et al., 2011). The interaction of *B. bifidum* with the host immune system has been assayed by investigating the impact of *B. bifidum* Z9 in combination with a second human gut commensal, *Lactobacillus acidophilus*, on the transcriptome of dendritic cells (DCs) (Weiss et al., 2010). This study highlighted that *B. bifidum* Z9 down-regulates the expression of genes involved to the adaptive immune system in murine DCs. Such findings corroborated other studies based on *in vitro* assays and involving various strains belonging to different *Bifidobacterium* species, which display a clear and distinct induction of cytokine profile by bifidobacteria. In particular, it was shown that *B. bifidum* strains, in contrast to representatives of other bifidobacterial species, provoked a significantly increased production of the IL-17 cytokine (Turroni et al., 2010; Lopez et al., 2011). The observation that *B. bifidum* strains induce an immune response affecting Treg/TH17 plasticity (Turroni et al., 2010; Lopez et al., 2011) leads to hypothesize that such commensal bacteria have a key role in mucosal tolerance, as also suggested by the demonstration that *B. bifidum*, differently from several other species of the genus, possesses the ability to induce IL-2 secretion by DCs (Guglielmetti et al., 2014a). In particular, it was demonstrated that the cell-surface-exposed molecule Tga of *B. bifidum* MIMBb75 is capable alone through its C-terminal CHAP domain of inducing DCs activation and IL-2 production (Guglielmetti et al., 2014b).

Recently, the host response triggered by the presence of *B. bifidum* PRL2010 cells was investigated thanks to a high-throughput gene expression technology and by utilizing both an *in vitro* cell line model as well as a murine model (Turroni et al., 2014a). Notably, the overall host-response scenario driven by *B. bifidum* PRL2010 cells can be described as a pro-inflammatory response priming the immune system, yet at the same time attenuating the pro-inflammatory response by down-regulation of certain chemokines, heat shock proteins (HSP) as well as stimulating the up-regulation of defensin and tight junction genes. In addition, results from ELISA experiments displayed that exposure to *B. bifidum* PRL2010 triggers the synthesis of IL-6 and IL-8 cytokines, presumably through NF-κb activation (Turroni et al., 2014a).

Other *in vivo* observations involving *B. bifidum* strains have been performed under chemically-induced diseases such as colitis. In this context, administration of *B. bifidum* strain S17 to mice with colitis was shown to suppress intestinal inflammation with a significant reduction in histology scores and the levels of pro-inflammatory cytokines interleukin IL-1β, IL-6, keratinocyte-derived chemokine and the inflammatory markers cyclooxygenase and myeloperoxidase (Philippe et al., 2011).

## *B. bifidum* STRAINS AS POTENTIAL HEALTH-PROMOTING CANDIDATES

Various strains of the *B. bifidum* species have been reported to exert health benefits to their human host, including antibacterial activities against pathogens such as *Helicobacter pylori* (Shirasawa et al., 2010; Chenoll et al., 2011), reduction of apoptosis in the intestinal epithelium of infants suffering from necrotizing enterocolitis (Khailova et al., 2010), modulation of the host-immune system (Fu et al., 2010; Philippe et al., 2011), and alleviation of anti-inflammatory activities associated with certain chronic large bowel dysfunctions (Mouni et al., 2009; Guglielmetti et al., 2011). In addition, *B. bifidum* together with other bifidobacterial species like *B. breve* and *B. longum* subsp. *infantis* are considered important for the establishment of a well-balanced, autochthonous intestinal microbiota in newborns (Tabbers et al., 2011). However, in order to exert a potential health-promoting activity in the human gut, bacteria need to reach this compartment in a viable form, while they should also be able to persist within the intestine. In this context, many *B. bifidum* strains have been shown to possess a strong adhesion phenotype to human epithelial intestinal cell monolayers (Caco-2 and HT29) (Guglielmetti et al., 2009; Serafini et al., 2013) and in a few cases, such as for *B. bifidum* PRL2010 and MIMBb75, were demonstrated to survive under gastrointestinal challenges (Serafini et al., 2013) and colonize the intestine impacting on the resident microbial communities at various intestinal loci (Singh et al., 2013). Another interesting phenotype displayed by probiotic bacteria is their displacement and competition against pathogens. Interestingly, *in vitro* trials based on HT29 monolayer involving *B. bifidum* PRL2010 cells displayed a clear inhibition of adhesion of pathogenic bacteria such as *Escherichia coli* and *Cronobacter sakazakii* (Serafini et al., 2013).

A health-promoting microorganism needs to be administered to its human host within a specific matrix in order to assure survival and biological functionality of its cells (e.g., producing probiotic molecules). Recently, kefir and kefiran were shown to affect the transcriptome of *B. bifidum* PRL2010 causing increased transcription of genes involved in the metabolism of dietary glycans as well as genes acting as host–microbe effector molecules such as pili (Serafini et al., 2014). Thus, the use of kefir and perhaps other (fermented) food products may be considered as a valuable means for the administration of *B. bifidum* cells to humans and may represent an effective food matrix to pre-adapt bifidobacterial cells to the host in order to enhance probiotic efficacy.

## INDUSTRIAL USE OF *B. bifidum*

Due to their health-promoting activities, much effort has been invested in the incorporation of bifidobacteria into probiotic food, supplements, and pharmaceutical preparations. By regulatory definition, microbial cells must be alive in a sufficient number in order to define a product as probiotic. In this perspective, the commercial use of *B. bifidum* as a probiotic has been limited by the fact that members of this species are particularly sensitive to stresses such as acidity and, in particular, oxygen (Jayamanne and Adams, 2006). Strategies to preserve probiotic cell viability are available and involve, for instance, microencapsulation (Zhang et al., 2013), and the addition of prebiotic molecules to the formulation (Guglielmetti et al., 2008a). However, these strategies do not overcome the problem of the limited biomass yields generally obtained in industrial fermentations due to the intrinsic stress sensitivity of *B. bifidum*, resulting in a strong increase of production costs. In this context, an aspect of particular importance is represented by strain “domestication.” In fact, once a bifidobacterial strain is isolated from its natural environment, its intrinsic sensitivity to oxygen decreases slowly and progressively during the numerous subcultivations under laboratory conditions. In this direction, an example is represented by strain *B. bifidum* MIMBb75, which was isolated about twelve years ago from a fecal sample of a healthy adult. Although originally very recalcitrant to laboratory cultivation, after hundreds of subculturings in aerobic atmosphere followed by incubation in a gas-pack with Anaerocult A, this strain drastically improved its ability to resist oxidative and other laboratory stresses (Guglielmetti S., personal communication). Thus, the selective pressure of a laboratory environment induced physiological changes that allowed the employment of strain *B. bifidum* MIMBb75 at industrial level; this strain, in fact, is now commercially available in a pharmaceutical probiotic product consisting of an encapsulated formulation, in which MIMBb75 cells can maintain a viability of at least 10^9^ CFU per capsule during the entire shelf-life of the product (Guglielmetti et al., 2011; Guglielmetti S., personal communication).

## CONCLUSION

**FIGURE 1:**
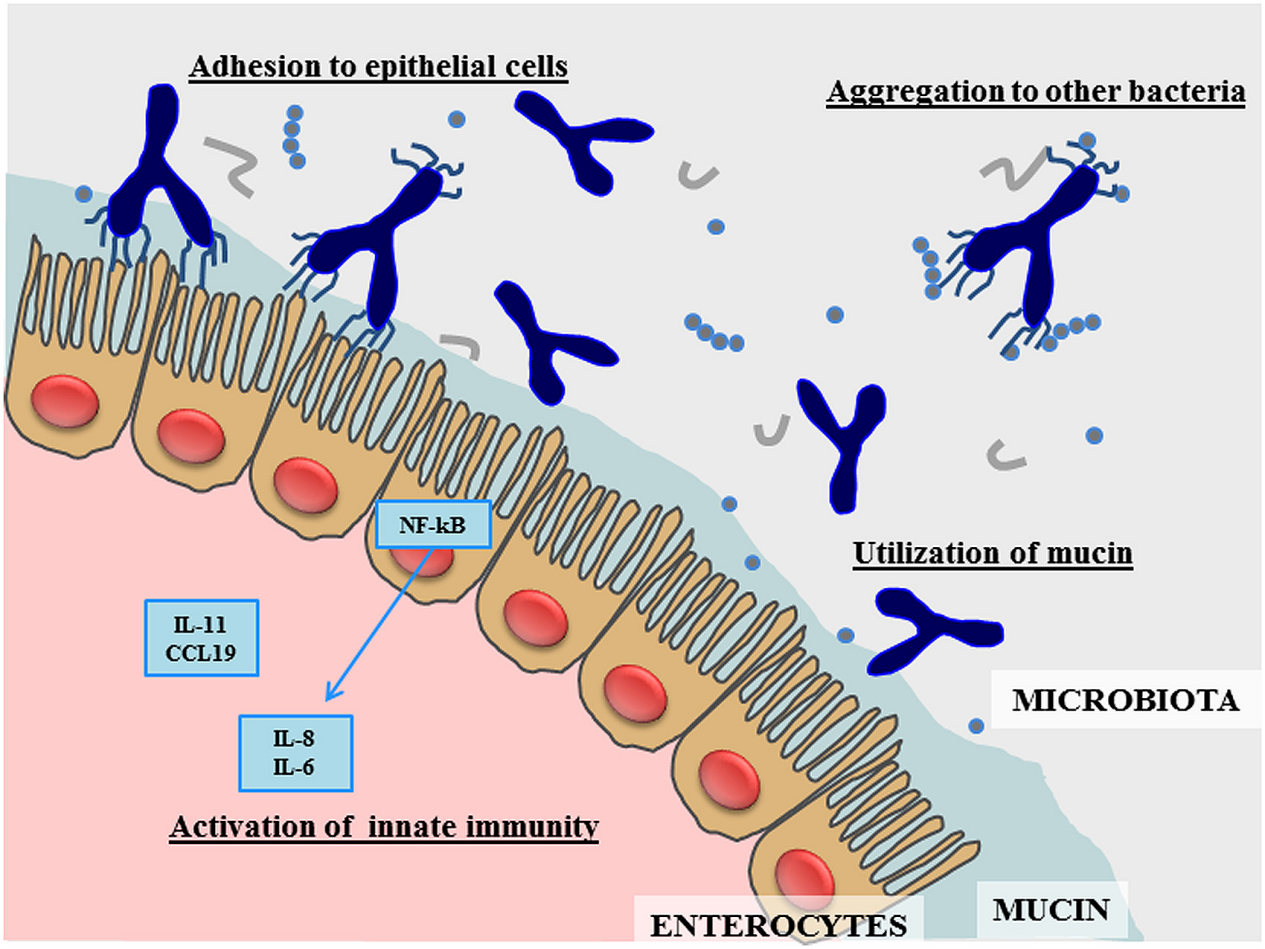
**Schematic representation of the main effects produced by *Bifidobacterium bifidum* in the human gut**.

During the last 5–10 years research in bifidobacteria has blossomed (Ventura et al., 2012, 2014; Turroni et al., 2014b). In this context, genomic investigations of bifidobacteria have revealed genetic repertoires that are considered crucial for conveying the typical saccharolytic phenotype of these bacteria, and which are worthy of detailed investigation for their potential roles in colonization of the human gut and their dependence on our diet. However, the discovery of the specific functional contribution to the host by each member of the bifidobacterial population in the human gut is still at its infancy. Furthermore, very little is known about the cross-talk that is believed to occur between individual members of the bifidobacterial microbiota (Egan et al., 2014) and with other members of the gut microbiota (Sonnenburg et al., 2006). The genetic data retrieved from the genome analysis of *B. bifidum* suggests the existence of various molecules that are responsible for specific health-promoting activities exerted by members of this bifidobacterial taxon (**Figure 1**). Future work needs to be carried out in order to better understand how these genetic features are exploited in the human gut through the use of high throughput metagenomic and post-genomic approaches. Nonetheless, currently available experimental data already supports the notion that *B. bifidum* represents a highly interesting bacterial species that is able to benefit human health in the prevention and treatment of gastrointestinal dysfunctions.

## Conflict of Interest Statement

The authors declare that the research was conducted in the absence of any commercial or financial relationships that could be construed as a potential conflict of interest.
